# Oxidative Stress: Dual Pathway Induction in Cardiorenal Syndrome Type 1 Pathogenesis

**DOI:** 10.1155/2015/391790

**Published:** 2015-03-04

**Authors:** Grazia Maria Virzì, Anna Clementi, Massimo de Cal, Alessandra Brocca, Sonya Day, Silvia Pastori, Chiara Bolin, Giorgio Vescovo, Claudio Ronco

**Affiliations:** ^1^Department of Nephrology, Dialysis and Transplant, San Bortolo Hospital, 36100 Vicenza, Italy; ^2^International Renal Research Institute of Vicenza (IRRIV), Italy; ^3^Department of Nephrology and Dialysis, San Giovanni Di Dio Hospital, 92100 Agrigento, Italy; ^4^Department of Medicine DIMED, University of Padua Medical School, 35128 Padua, Italy; ^5^Laboratory of Experimental Hepatology, Department of Medicine, University of Padua, 35128 Padua, Italy; ^6^Department of Information Engineering, University of Padua, 35128 Padua, Italy; ^7^Internal Medicine, San Bortolo Hospital, 36100 Vicenza, Italy; ^8^Internal Medicine Unit, Sant'Antonio Hospital, 35127 Padua, Italy

## Abstract

Cardiorenal Syndrome Type 1 (Type 1) is a specific condition which is characterized by a rapid worsening of cardiac function leading to acute kidney injury (AKI). Even though its pathophysiology is complex and not still completely understood, oxidative stress seems to play a pivotal role. In this study, we examined the putative role of oxidative stress in the pathogenesis of CRS Type 1. Twenty-three patients with acute heart failure (AHF) were included in the study. Subsequently, 11 patients who developed AKI due to AHF were classified as CRS Type 1. Quantitative determinations for IL-6, myeloperoxidase (MPO), nitric oxide (NO), copper/zinc superoxide dismutase (Cu/ZnSOD), and endogenous peroxidase activity (EPA) were performed. CRS Type 1 patients displayed significant augmentation in circulating ROS and RNS, as well as expression of IL-6. Quantitative analysis of all oxidative stress markers showed significantly lower oxidative stress levels in controls and AHF compared to CRS Type 1 patients (*P* < 0.05). This pilot study demonstrates the significantly heightened presence of dual oxidative stress pathway induction in CRS Type 1 compared to AHF patients. Our findings indicate that oxidative stress is a potential therapeutic target, as it promotes inflammation by ROS/RNS-linked pathogenesis.

## 1. Introduction

Heart performance and kidney function are strictly interconnected and communication between these two organs occurs through a variety of pathways, including hemodynamic and nonhemodynamic mechanisms [1–6]. Heart and kidney disease often coexist in the same patient in acute and chronic states. Clinical trial data have indeed demonstrated that cardiac disease can directly contribute to worsening kidney function and vice versa. These critical, dynamic, and bidirectional connections between both acute and chronic cardiac dysfunction and acute and chronic kidney disease are well recognized and have been defined as cardiorenal syndromes (CRS) by the consensus conference of Acute Dialysis Quality Initiative (ADQI) [3, 4]. The current definition has been expanded into 5 subtypes whose etymology reflects the primary and secondary pathology, the time frame, and the simultaneous cardiac and renal codysfunction secondary to systemic disease [2, 4].

A large body of data indicates that the bidirectionality and the temporal pattern of cellular and humoral signaling between these two organs occurs through a variety of mechanisms [6], including oxidative damage, sustained cell activation, metabolic dysregulation and inflammation leading to monocyte phenotype transition, myocyte apoptosis, and activation of matrix metalloproteinases [3, 5, 7–17].

Cardiorenal Syndrome Type 1 (CRS Type 1) is a specific condition which is characterized by a rapid worsening of cardiac function leading to acute kidney injury (AKI). CRS Type 1 occurs in approximately 25% to 33% of patients with acute decompensate heart failure (ADHF) and represents an important consequence of hospitalization with a myriad of implications in terms of diagnosis, management, prognosis, and cost of care [5, 18–21].

The pathophysiology of CRS Type 1 is complex and it is not still completely understood. Oxidative stress seems to play a pivotal role in the pathogenesis of this syndrome. Oxidative loss of redox homeostasis in reactive oxygen species (ROS) and reactive nitrogen species (RNS) results, indeed, in an immune system activation and in a proinflammatory and profibrotic milieu via distinct mechanisms which stimulate renal and cardiovascular structural and functional abnormalities [5, 22–25]. Although physiological levels of ROS are necessary for a normal cellular function, the overproduction of these molecules is responsible for both cardiac dysfunction and renal dysfunction. Thus, therapeutic attempts to substantially attenuate oxidative stress, in theory, hold promise for large benefits in patients with CRS Type 1 [3, 21].

In this study, we examined the putative role of ROS and RNS in the pathogenesis of CRS Type 1. We evaluated IL-6 and the oxidative stress levels, by measuring myeloperoxidase and endogenous peroxidase activity and quantifying nitric oxide and copper/zinc superoxide dismutase levels in our study population.

## 2. Materials and Methods

### 2.1. Study Population

Patients admitted to the Internal Medicine Department of San Bortolo Hospital in Vicenza, Italy, between September 2011 and December 2011 were screened. A total of 40 patients with acute heart failure (AHF) were further examined for inclusion into the study. Patients with acute kidney injury (AKI) prior to the episode of AHF, patients with other potential causes of AKI or patients with estimated glomerular filtration rate (eGFR) < 45 mL/min/1.73 m^2^ (CKD stage 3a), and patients with previous kidney transplantation were excluded. Septic patients and hypotensive patients who required inotropic support prior to the diagnosis of AKI were not included into the study. We considered, as the baseline value, the creatinine level of the 3 months before the admission of all the patients enrolled into the study.

Twenty-three patients were finally enrolled. Subsequently, 11 patients who developed AKI due to acute heart failure during the course of hospitalization were classified as CRS Type 1. Acute kidney injury was presumed to be related to cardiac dysfunction after having excluded other possible causes of renal damage based on the review of the clinical course of the patients. The 12 patients with AHF who did not develop AKI during hospitalization were analysed to better understand the contribution of cardiac dysfunction on oxidative stress.

Clinical data, blood pressure, serum creatinine (SCr), blood urea, haemoglobin, serum albumin, brain natriuretic peptide (BNP), aspartate aminotransferase (AST), alanine aminotransferase (ALT), lactic acid dehydrogenase (LDH), and creatine phosphokinase (CPK) were evaluated and collected at admission. Echocardiograms were performed within 6 hours from the admission into the Internal Medicine ward. AHF was defined by the European Society of Cardiology (ESC) guidelines [26]. AKI was defined by the Acute Kidney Injury Network (AKIN) criteria [27]. SCr was measured by Jaffè-Method and the eGFR was calculated with the 4-variable standardized-MDRD study equations. CRS Type 1 was defined according to the current classification system [2–4]. Hypertension was defined according to the European Society of Cardiology (ESC) guidelines for the management of arterial hypertension (normal range: systolic blood pressure (SBP) < 130–139 mmHg and diastolic blood pressure (DBP) < 85–89 mmHg) [28]. Obesity was defined by the OMS classification (normal range of Body Mass Index 18.5 < (BMI) < 24.9) [29–31]. Diabetes was defined according to the American Diabetes Association (ADA) guidelines [32].

All the procedures were in accordance with the Helsinki Declaration. The protocol and consent form were approved by the Ethics Committee of San Bortolo Hospital. All the patients were informed about the experimental protocol and the objectives of the study before providing informed consent and blood samples.

In addition, 15 healthy volunteers without AHF or AKI were recruited as control group for this study (CTR).

### 2.2. Sample Collection

Peripheral venous blood samples were collected from all 23 patients within 8 hours from the admission into the Internal Medicine ward. We also collected blood sample within 24 h of AKI for patients who developed CRS Type 1. The blood samples were collected in EDTA tubes and subsequently centrifuged for 10 minutes at 3500 rpm. After centrifugation, plasma was immediately separated from blood cells and stored at −80°C. All the samples were processed within 4 hours after collection.

### 2.3. IL-6 Enzyme-Linked Immunosorbent Assay (ELISA)

Quantitative determination of IL-6 in plasma samples was performed by Human Instant ELISA kit (eBioscience, San Diego, CA, USA).

Cytokine determination was performed according to manufacturer's protocol and instructions. Optical density was read by using a VICTORX4 Multilabel Plate Reader (PerkinElmer Life Sciences, Waltham, MA, USA) at 450 nm. The levels of this molecule were calculated from standard curves, according to the manufacturer's protocol. All the tests were performed in triplicate. Standard samples for IL-6 ranged from 3.1 to 200 ng/mL and the sensitivity of this test was 0.92 ng/mL.

### 2.4. Oxidative Stress Detection

A quantitative determination of oxidative stress was performed in the plasma samples of the patients with acute heart failure, CRS Type 1.

#### 2.4.1. Myeloperoxidase (MPO) ELISA Detection

Quantitative determination of plasma MPO concentration was performed by Human Instant ELISA kit (eBioscience, San Diego, CA, USA).

Preliminary plasma dilution 1 : 100 was performed for each sample with Sample Diluent (eBioscience, San Diego, CA, USA). MPO determination was performed according to the manufacturer's protocol and instructions. Optical density was read by using a VICTORX4 Multilabel Plate Reader (PerkinElmer Life Sciences, Waltham, MA, USA) at 450 nm. The levels of this molecule were calculated from the standard curve according to the manufacturer's protocol. Standard samples ranged from 0.16 to 10.0 ng/mL. Human MPO Instant ELISA Kit sensitivity is 0.03 pg/mL. All tests were performed in triplicate.

#### 2.4.2. Colorimetric Assay for Nitric Oxide (NO) Quantification

Immunoaffinity purified nitrate reductase (NaR) enables the measurement of the total nitric oxide (NO) produced in in vitro experimental systems. Nitric oxide can be spectrophotometrically assayed by measuring the accumulation of its stable degradation products, nitrate and nitrite. Quantitative determination of NO concentration in plasma samples was performed by Nitric Oxide Colorimetric Assay Kit (Oxford Biomedical Research, Aachen, Germany). NO determination was performed according to the manufacturer's protocol and instructions. Optical density was read by using a VICTORX4 Multilabel Plate Reader (PerkinElmer Life Sciences, Waltham, MA, USA) at 540 nm. The levels of these molecules were calculated from the standard curve according to the manufacturer's protocol. Standard samples ranged from 0.5 to 100.0 *µ*M. All the tests were performed in triplicate.

#### 2.4.3. Copper/Zinc Superoxide Dismutase (Cu/ZnSOD) ELISA Detection

Quantitative determination of Cu/ZnSOD concentration in plasma samples was performed by Human ELISA kit (eBioscience, San Diego, CA, USA).

Preliminary plasma dilution 1 : 20 was performed for each sample with Sample Diluent (eBioscience, San Diego, CA, USA). Cu/ZnSOD determination was performed according to the manufacturer's protocol and instructions. Optical density was read by using a VICTORX4 Multilabel Plate Reader (PerkinElmer Life Sciences, Waltham, MA, USA) at 450 nm. The levels of these molecules were calculated from the standard curve according to the manufacturer's protocol. Standard samples ranged from 0.08 to 5.0 ng/mL. Human MPO Instant ELISA Kit sensitivity is 0.04 ng/mL. All the tests were performed in triplicate.

#### 2.4.4. Endogenous Peroxidase Activity (EPA) Quantification

EPA is a colorimetric test for the quantitative determination of endogenous peroxidase activity in EDTA-plasma. The determination of the endogenous peroxidase activity is based on the reaction of peroxides with peroxidase followed by a color reaction of the chromogenic substrate tetramethylbenzidine.

Its blue color turns to yellow after the addition of the stop solution, and it can be measured photometrically at 450 nm by VICTORX4 Multilabel Plate Reader (PerkinElmer Life Sciences, Waltham, MA, USA). EPA quantifications were performed according to the manufacturer's protocol and instructions. Quantification was achieved by serial dilutions of a standard peroxidase solution. The calibration curve was obtained by plotting the extinction values measured for the 5 standards against the corresponding concentrations (0, 5, 10, 15, and 20 U/L). The results can be calculated using a linear fit. Each sample was performed in triplicate.

### 2.5. Statistical Analysis

Statistical analysis was performed using the STATA Software package. Categorical variables were expressed as percentages; continuous variables were expressed as means ± standard deviation (parametric variables) or median and interquartile range (IQR) (nonparametric variables).

Comparisons of the two cohorts were made using a chi-square test or Fisher's exact test for categorical variables and a Mann-Whitney *U* test or *t*-test for continuous variables, as appropriate. The Kruskal-Wallis test for multiple comparisons was applied to compare the groups. A *P* value of <0.05 was considered statistically significant.

## 3. Results

### 3.1. Subjects Characteristics

Acute heart failure, defined by the European Society of Cardiology (ESC) guidelines [26], was caused by non-ST segment elevation myocardial infarction in 8.7% of patients, excessive salt and fluid intake in 39.1% of patients, hypertensive crisis in 21.7% of patients, and other causes in 17.4% of patients. In 13.1% of patients, no cause of acute heart failure was recognized.

The mean age of 11 patients with CRS Type 1 was 76 ± 10 years and 54% of the patients were males. The median baseline SCr of CRS Type 1 patients was 0.96 mg/dL (IQR 0.88–1.02), and the median eGFR was 62 mL/min/1.73 m^2^ (IQR 55–75). Seven (63%) CRS Type 1 subjects had diabetes mellitus and 10 (90%) had hypertension. Three patients (27%) developed AKI during the first day of hospitalization, 6 (55%) patients developed it during the second day, and 2 patients (18%) developed it during the third day. All CRS Type 1 patients were classified in AKIN stage 1. The mean age of 12 patients with AHF was 80 ± 8 years and 58% of these patients were males. The median baseline SCr of AHF subjects was 0.98 mg/dL (IQR 0.87–1.15), and the median eGFR was 67 mL/min/1.73 m^2^ (IQR 53–82). 5 (42%) AHF subjects had diabetes mellitus and 11 (92%) AHF subjects had hypertension. Characteristics of CRS Type 1 and AHF patients are described in Table 1. Medications of CRS Type 1 and AHF patients are described in Table 2. 

No patients were exposed to radiocontrast media in the 72 hours preceding AKI. No patients developed the need of mechanical ventilation and renal replacement therapy (RRT). Urea, haemoglobin, serum albumin, BNP, and Troponin I levels were not significantly different at admission in CRS Type 1 and AHF patients. Medication treatments were similar in CRS Type 1 and AHF patients. In particular, the amount of diuretics administered was similar in these two groups.

### 3.2. Inflammatory Cytokine and Markers of Oxidative Stress

IL-6 levels were significantly elevated both in AHF and in CRS type 1, when compared with CTR (5.9 pg/mL, IQR 3.4–7.6) (both *P* < 0.01). In addition, IL-6 levels were significantly higher in patients with CRS Type 1 when compared with patients with AHF. Also MPO, NO, Cu/Zn SOD, and EPA levels were significantly higher both in AHF and in CRS Type 1 patients, when compared with CTR. Furthermore, all oxidative stress markers were significantly elevated in CRS Type 1 patients compared with AHF patients (*P* < 0.05) (Table 3). Specifically, the median values of IL-6 were 5 times higher in CRS type 1 patients compared with AHF subjects. Similarly, the median MPO and Cu/Zn SOD concentrations were 1.5 times higher in the CRS Type 1 group. Furthermore, the median NO levels were 2 times higher in CRS Type 1 patients compared with AHF subjects, and the median EPA levels were 10 times higher in the first group.

**Figure 1:**
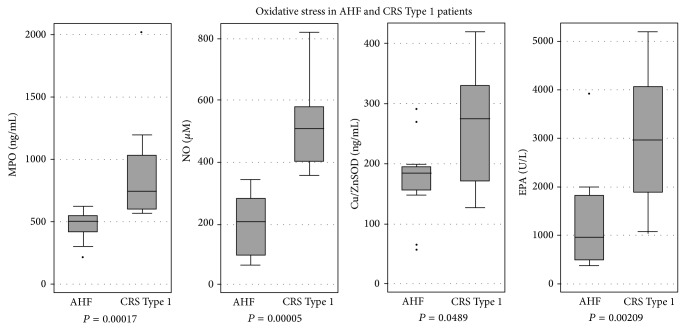


Our results demonstrate the significantly heightened presence of dual reactive oxygen species imbalance in patients with CRS Type 1 compared to AHF patients: ROS/RNS production involving NADPH oxidase and MPO; SOD production of hydrogen peroxide; and NO upregulation of proinflammatory mediators via peroxynitrite (Figure 1).

## 4. Discussion

Oxidative stress is a common pathway involved in cellular dysfunction, tissue injury, and organ failure and it is defined as a result of an imbalance between oxidants and antioxidants molecules in favour of the former [33]. Oxidative stress occurs when the formation of ROS exceeds the body's ability to metabolize them or when the antioxidant defence mechanisms are depleted. ROS are oxygen-derived small molecules, including oxygen radicals' superoxide, hydroxyl, peroxyl, alkoxyl, and nonradicals, such as hydrogen peroxide (H_2_O_2_) [17]. There are many cellular sources of ROS, such as mitochondria, NADH/NADPH oxidases, response to cytokines, and other growth factors receptors [34]. High levels of oxygen radicals inactivate mitochondrial enzymes, cause DNA damage, induce base hydroxylation, and strand breaks, thus leading to cell injury and apoptosis [35].

In addition, ROS production can lead to “ROS-induced ROS release,” a vicious circle in which ROS species activate the permeability of mitochondrial pores leading to mitochondrial dysfunction and to further ROS release [36]. ROS are known to activate NF-kappaB which in turn activates growth factors and antiapoptotic molecules resulting in cell proliferation (cancer), inflammatory cytokines, and adhesion molecules [37].

The enzymatic detoxification mechanisms involve a number of antioxidant enzymes (superoxide dismutases, catalases, glutathione peroxidases, and peroxiredoxins), small molecular-weight antioxidants, and adaptive mechanism leading to antioxidant gene expression [38].

Antioxidant systems can stabilize free radicals, consequently reducing the oxidative stress. Enzymatic antioxidants are the most important defense against radical-induced damage [39].

Our results demonstrate a significant increase in both ROS and RNS redox disequilibrium in patients with CRS Type 1 compared to patients with acute heart failure and control subjects. In particular, CRS Type 1 patients presented a significant increase in circulating ROS and RNS, as well as an increased expression of inflammatory cytokines, in particular that of IL-6. Increased levels of NADPH oxidase and MPO as well as SOD upregulation with concomitant upregulation of proinflammatory mediators via peroxynitrite have not been previously reported. MPO is a haeme enzyme that is abundant in granules of human inflammatory cells such as activated neutrophils, macrophages, and monocytes. MPO acts as a master enzyme in the generation of a range of ROS by catalyzing the conversion of hydrogen peroxide (H_2_O_2_) into species including OH, ONOO, hypochlorous acid (HOCl), and NO_2_. MPO-catalyzed species are involved further in oxidative damage of various biological molecules (e.g., lipids, lipoproteins, and proteins low) and tissue degradation and are implicated in atherosclerosis, cancer, diabetic vascular complications, kidney diseases, and other disorders [40–43]. In prospective studies, high MPO levels were able to predict increased risk of developing CAD in healthy individuals [44] and cardiovascular events in patients presenting to emergency with chest pain [45] and increased risk of myocardial infarction and death in patients with acute coronary syndrome [46]. Furthermore, MPO has been speculated to be a major oxidative stress pathway in ESRD [47].

Furthermore, it is known that superoxide production of hydrogen peroxide and nitric oxide upregulation is responsible for an increase in IL-6 production and secretion. In our study, IL-6 resulted to be higher in patients with CRS Type 1 who presented higher levels of oxidative stress markers.

Increased levels of SOD have not been previously observed in CRS Type 1 patients. SODs are a unique family of metalloproteins that catalytically enhances the normal dismutation of superoxide. SOD is normally present at low micromolar concentrations in cells. Four types of SOD have been defined on the basis of distinctions in their metal cofactors and distribution: manganese (MnSOD) principally located in the matrix of mitochondria of all aerobes, copper/zinc (Cu/ZnSOD) mainly present in the cytoplasm of eukaryotic cells, iron (FeSOD) predominantly present in the cytosol, chloroplasts, or mitochondria of prokaryotes, and extracellular (ECSOD) found in the extracellular fluids or membrane associated in mammals [48–50]. In particular, we noted a specific activity of Cu/ZnSOD in CRS Type 1 patients.

Increased ROS production has been implicated in many pathological conditions, such as hypertension, diabetes mellitus, hypercholesterolemia, restenosis, heart failure, kidney diseases, and atherosclerosis [51–55]. Specifically, atherosclerosis results from a local imbalance between ROS productions, leading to oxidative stress and these antioxidant enzymes [56].

Both in vitro and animal studies have demonstrated that several pathways are dysregulated in heart failure, leading to increased oxidative stress markers production and cardiac damage. A metabolic shift from fatty acid (FA) oxidation to glycolysis has been indeed reported in cardiomyocytes in the setting of heart failure. Myocardial ATP content gradually decreases, dropping to 60%–70% of normal levels [57–59]. This drop is due to a decrease in mitochondrial oxidative metabolism and it is balanced by a compensatory increase in glucose uptake and in glycolysis [60, 61]. The reduced oxidative metabolism leads to an accumulation of free FA in cardiomyocytes, creating a self-perpetuating mechanism of ever-increasing oxidative stress responsible for deleterious effects on the heart.

Once produced, ROS display several negative effects on cardiac cells, impairing cardiomyocyte contractility, ion transport, and calcium handling. In addition to their detrimental effects, mitochondrial ROS play an important role in intracellular signalling by triggering multiple cellular pathways and the transcriptional activation of selected nuclear genes, finally eliciting transcriptional reprogramming [62, 63]. We speculated a similar cardiac condition in CRS Type 1 patients: the higher redox disequilibrium observed in these patients, compared to AHF subjects, could be involved in renal damage. We observed a significant difference in oxidative response between CRS Type 1 patients and AHF patients. Although these findings are provocative, the design of the study does not allow us to make conclusions about causality. Indeed, increased oxidative stress could be secondary to the renal injury rather than the cause of this complication. Further studies are needed to support this hypothesis.

Within the kidneys, ROS generation increases in response to specific stimuli, such as angiotensin II and aldosterone secretion [17]. NOX enzymes are the primary source of ROS in vascular smooth cells in both kidney cortex and medulla [64, 65]. Under physiological conditions, NO induces vasodilatation of the afferent arteriole, thus increasing renal blood flow, blunts tubule-glomerular feedback, promotes pressure natriuresis, and scavenges low ROS concentrations [66]. In case of increased oxidative stress, superoxide production leads to cascade reactions which result in vasoconstriction, inflammation, and impaired vascular and renal functions [17]. In fact, we observed a significant increase in circulating oxidative stress species and an increased expression of IL-6 in CRS Type 1 patients. In fact, we observed a stronger IL-6 and inflammation activation in CRS Type 1 patients compared to AHF subjects.

In CRS Type 1 group, diabetes was more frequent than in AHF population. It is well known that hyperglycemia results in excessive production of acetyl-CoA that feeds into the Krebs cycle, thus increasing NADH production [67]. Therefore, oxidation of the overproduced NADH by mitochondria inevitably leads to the production of more superoxide and hence more ROS [68]. This is responsible for the accumulation of glycolytic metabolites upstream of glyceraldehydes 3-phosphate and the activation of the alternative glucose disposal pathways that all are linked to ROS production. Even though in our study the percentage of diabetic patients was not significantly different between the two groups, maybe because of the small size of the sample, the presence of diabetes may have increased the oxidative stress in CRS Type 1 patients.

The critical roles of inflammation and immune system dysregulation in the CRS Type 1 pathophysiology have been reported both in animal and human models which indicate that proinflammatory cytokines and chemokines are associated with molecular, clinical, and physiological aspects of this syndrome [5, 69–72]. Cellular metabolic shifts in response to humoral and cellular signaling factors may result in an upregulated expression and release of proinflammatory/immune-modulatory cytokines, which are released into the renal tissue and in the blood, respectively [69, 73–75]. In this pilot study, we observed the inflammatory process activation and the loss of redox homeostasis in CRS Type 1. These observations indicate that cellular responses to different signals influence a dual shift in both ROS and RNS production.

## 5. Conclusion

These preliminary results underline the importance of oxidative stress in the pathogenesis of CRS Type 1. Given the myriad of implications of this syndrome in terms of diagnosis, management, prognosis, and cost of care, understanding the mechanism by which inflammatory cascades are activated as a results of oxidative stress has important clinical implications.

This study explores the premise of ROS/RNS disequilibrium in the CRS Type 1 pathophysiology. Nevertheless, we acknowledge the limitations of the small sample size in this pilot study, which would preclude meaningful multivariate analysis. Our preliminary results can be considered as hypothesis-generating about CRS Type 1 pathogenesis, allowing further exploration of novel pathophysiological mechanisms in CRS Type 1. Further studies are needed to better understand the role that these molecules and therapeutics have in altering target processes.

## Figures and Tables

**Table 1:** Baseline characteristics of CRS Type 1 and AHF patients and clinical parameters.

	AHF	CRS Type 1	*P* value
Age, years	80.0 ± 8.0	76.0 ± 13.0	NS
Weight, Kg	75 (64–88)	77 (67–85)	NS
Diabetes	42%	64%	NS
Hypertension	92%	91%	NS
Peripheral vascular disease	42%	45%	NS
Cardiovascular disease	17%	18%	NS
Obesity	25%	27%	NS
Dyslipidemia	42%	45%	NS
Creatinine, mg/dL	0.98 (0.87–1.15)	0.96 (0.88–1.02)	NS
eGFR, mL/min/1.73 m^2^	67 (53–82)	62 (55–75)	NS
Mean arterial pressure (MAP), mm/Hg	103.3 (93.3–120.8)	100 (89.2–115.8)	NS
Ejection fraction	35% (24.0–48.0)	35% (25.0–51.0)	NS
BNP, pg/mL	632 (398.3–946)	695 (408.5–1837)	NS
Troponin I, ng/mL	0.07 (0.04–0.27)	0.07 (0.04–0.26)	NS
Hemoglobin, g/dL	11.1 (13.6–14.25)	11.4 (9.7–13)	NS
Albumin, g/L	4.01 (4.18–4.4)	4.3 (3.96–4.4)	NS
AST, U/L	22 (13.5–30.5)	21 (18.8–25.3)	NS
ALT, U/L	16 (13–30.5)	19 (17.3–25)	NS
LDH, U/L	367 (340–462)	435 (382–621)	NS
CPK, U/L	88 (48.5–115)	66.5 (38.5–83.5)	NS

Values denote means ± SD or medians (IQR) unless specified otherwise.

**Table 2:** Medication of CRS Type 1 and AHF patients.

	AHF	CRS Type 1	*P* value
Angiotensin-converting-enzyme inhibitor (ACEi)	58%	55%	NS
Angiotensin II receptor blockers (ARB)	17%	18%	NS
*β*-blocker	66%	63%	NS
Calcium antagonist	25%	27%	NS
Diuretics	92%	100%	NS
Statines	50%	55%	NS
Nonsteroidal anti-inflammatory drugs (NSAIDs)	8%	9%	NS

**Table 3:** Oxidative stress and IL-6 levels in AHF, CRS Type 1 patients, and CTR.

	AHF	CRS Type 1	CTR	*P* value
MPO, pg/mL	505.6 (421.7–547.8)	746.9 (665.2–940.0)	10.1 (6.0–19.3)	<0.01
NO, *µ*M	205.6 (95.0–277.5)	507.3 (404.7–557.3)	9.5 (6.1–12.2)	<0.01
Cu/ZnSOD, pg/mL	184.5 (160.5–192.0)	274.5 (191.8–326.8)	58.9 (51.7–70.9)	<0.01
EPA, U/L	274.5 (191.8–326.8)	2978.4 (2071.8–4069.9)	2.0 (0.9–3.9)	<0.01
IL-6, pg/mL	22.19 (16.6–24.6)	90.68 (59.9–105.3)	5.9 (3.4–7.6)	<0.01

Values denote medians (IQR).

## References

[1] Clementi A., Virzi G. M., Goh C. Y. (2013). Cardiorenal syndrome type 4: a review. *Cardiorenal Medicine*.

[2] Ronco C., Chionh C.-Y., Haapio M., Anavekar N. S., House A., Bellomo R. (2009). The cardiorenal syndrome. *Blood Purification*.

[3] Ronco C., Cicoira M., McCullough P. A. (2012). Cardiorenal syndrome type 1: pathophysiological crosstalk leading to combined heart and kidney dysfunction in the setting of acutely decompensated heart failure. *Journal of the American College of Cardiology*.

[4] Ronco C., Haapio M., House A. A., Anavekar N., Bellomo R. (2008). Cardiorenal syndrome. *Journal of the American College of Cardiology*.

[5] Virzì G. M., Day S., de Cal M., Vescovo G., Ronco C. (2014). Heart-kidney crosstalk and role of humoral signaling in critical illness. *Critical Care*.

[6] Viswanathan G., Gilbert S. (2011). The cardiorenal syndrome: making the connection. *International Journal of Nephrology*.

[7] Kinsey G. R., Li L., Okusa M. D. (2008). Inflammation in acute kidney injury. *Nephron—Experimental Nephrology*.

[8] Lee D. W., Faubel S., Edelstein C. L. (2011). Cytokines in acute kidney injury (AKI). *Clinical Nephrology*.

[9] Mangan D. F., Wahl S. M. (1991). Differential regulation of human monocyte programmed cell death (apoptosis) by chemotactic factors and pro-inflammatory cytokines. *Journal of Immunology*.

[10] Murakami M., Hirano T. (2012). The pathological and physiological roles of IL-6 amplifier activation. *International Journal of Biological Sciences*.

[11] Nian M., Lee P., Khaper N., Liu P. (2004). Inflammatory cytokines and postmyocardial infarction remodeling. *Circulation Research*.

[12] Satoh M., Ishikawa Y., Itoh T., Minami Y., Takahashi Y., Nakamura M. (2008). The expression of TNF-alpha converting enzyme at the site of ruptured plaques in patients with acute myocardial infarction. *European Journal of Clinical Investigation*.

[13] Schindler R., Boenisch O., Fischer C., Frei U. (2000). Effect of the hemodialysis membrane on the inflammatory reaction in vivo. *Clinical Nephrology*.

[14] Torre-Amione G. (2005). Immune activation in chronic heart failure. *The American Journal of Cardiology*.

[15] Wrigley B. J., Lip G. Y. H., Shantsila E. (2011). The role of monocytes and inflammation in the pathophysiology of heart failure. *European Journal of Heart Failure*.

[16] Metra M., Cotter G., Gheorghiade M., Cas L. D., Voors A. A. (2012). The role of the kidney in heart failure. *European Heart Journal*.

[17] Rubattu S., Mennuni S., Testa M. (2013). Pathogenesis of chronic cardiorenal syndrome: is there a role for oxidative stress?. *International Journal of Molecular Sciences*.

[18] Hata N., Yokoyama S., Shinada T. (2010). Acute kidney injury and outcomes in acute decompensated heart failure: evaluation of the RIFLE criteria in an acutely ill heart failure population. *European Journal of Heart Failure*.

[19] Liang K. V., Williams A. W., Greene E. L., Redfield M. M. (2008). Acute decompensated heart failure and the cardiorenal syndrome. *Critical Care Medicine*.

[20] Ronco C., McCullough P. A., Anker S. D. (2010). Cardiorenal syndromes: an executive summary from the consensus conference of the acute dialysis quality initiative (ADQI). *Contributions to Nephrology*.

[21] Cruz D. N. (2013). Cardiorenal syndrome in critical care: the acute cardiorenal and renocardiac syndromes. *Advances in Chronic Kidney Disease*.

[22] Gill R., Tsung A., Billiar T. (2010). Linking oxidative stress to inflammation: toll-like receptors. *Free Radical Biology and Medicine*.

[23] Khan S. R. (2013). Stress oxidative: nephrolithiasis and chronic kidney diseases. *Minerva Medica*.

[24] Li P.-L., Zhang Y. (2013). Cross talk between ceramide and redox signaling: Implications for endothelial dysfunction and renal disease. *Handbook of Experimental Pharmacology*.

[25] Mandavia C. H., Aroor A. R., Demarco V. G., Sowers J. R. (2013). Molecular and metabolic mechanisms of cardiac dysfunction in diabetes. *Life Sciences*.

[26] McMurray J. J., Adamopoulos S., Anker S. D. (2012). ESC guidelines for the diagnosis and treatment of acute and chronic heart failure 2012: The Task Force for the Diagnosis and Treatment of Acute and Chronic Heart Failure 2012 of the European Society of Cardiology. Developed in collaboration with the Heart Failure Association (HFA) of the ESC. *European Journal of Heart Failure*.

[27] Mehta R. L., Kellum J. A., Shah S. V. (2007). Acute Kidney Injury Network: report of an initiative to improve outcomes in acute kidney injury. *Critical Care*.

[28] Volpe M., Toccib G. (2009). 2007ESH/ESC Guidelines for the management of hypertension, from theory to practice: global cardiovascular risk concept. *Journal of Hypertension*.

[29] Haslam D. W., James W. P. T. (2005). Obesity. *The Lancet*.

[30] Berke E. M., Morden N. E. (2000). Medical management of obesity. *American Family Physician*.

[31] Galtier F. (2010). Definition, epidemiology, risk factors. *Diabetes & Metabolism*.

[32] American Diabetes Association (2010). Diagnosis and classification of diabetes mellitus. *Diabetes Care*.

[33] Sies H. (1997). Oxidative stress: oxidants and antioxidants. *Experimental Physiology*.

[34] Morgan M. J., Liu Z.-G. (2011). Crosstalk of reactive oxygen species and NF-kappaB signaling. *Cell Research*.

[35] Naziroğlu M., Yoldaş N., Uzgur E. N., Kayan M. (2013). Role of contrast media on oxidative stress, Ca^2+^ signaling and apoptosis in kidney. *Journal of Membrane Biology*.

[36] Maack C., Böhm M. (2011). Targeting mitochondrial oxidative stress in heart failure: throttling the afterburner. *Journal of the American College of Cardiology*.

[37] Lin Y., Bai L., Chen W., Xu S. (2010). The NF-*κ*B activation pathways, emerging molecular targets for cancer prevention and therapy. *Expert Opinion on Therapeutic Targets*.

[38] Kalyanaraman B. (2013). Teaching the basics of redox biology to medical and graduate students: oxidants, antioxidants and disease mechanisms. *Redox Biology*.

[39] Sung C.-C., Hsu Y.-C., Chen C.-C., Lin Y.-F., Wu C.-C. (2013). Oxidative stress and nucleic acid oxidation in patients with chronic kidney disease. *Oxidative Medicine and Cellular Longevity*.

[40] Arnhold J. (2004). Properties, functions, and secretion of human myeloperoxidase. *Biochemistry (Moscow)*.

[41] Reeves E. P., Nagl M., Godovac-Zimmermann J., Segal A. W. (2003). Reassessment of the microbicidal activity of reactive oxygen species and hypochlorous acid with reference to the phagocytic vacuole of the neutrophil granulocyte. *Journal of Medical Microbiology*.

[42] Borawski J. (2006). Myeloperoxidase as a marker of hemodialysis biocompatibility and oxidative stress: the underestimated modifying effects of heparin. *The American Journal of Kidney Diseases*.

[43] Ho E., Karimi Galougahi K., Liu C.-C., Bhindi R., Figtree G. A. (2013). Biological markers of oxidative stress: applications to cardiovascular research and practice. *Redox Biology*.

[44] Meuwese M. C., Stroes E. S. G., Hazen S. L. (2007). Serum myeloperoxidase levels are associated with the future risk of coronary artery disease in apparently healthy individuals: the EPIC-Norfolk Prospective Population Study. *Journal of the American College of Cardiology*.

[45] Brennan M.-L., Penn M. S., Van Lente F. (2003). Prognostic value of myeloperoxidase in patients with chest pain. *The New England Journal of Medicine*.

[46] Baldus S., Heeschen C., Meinertz T. (2003). Myeloperoxidase serum levels predict risk in patients with acute coronary syndromes. *Circulation*.

[47] Maruyama Y., Lindholm B., Stenvinkel P. (2004). Inflammation and oxidative stress in ESRD—the role of myeloperoxidase. *Journal of Nephrology*.

[48] Barra D., Martini F., Bannister J. V. (1980). The complete amino acid sequence of human Cu/Zn superoxide dismutase. *FEBS Letters*.

[49] Shull S., Heintz N. H., Periasamy M. (1991). Differential regulation of antioxidant enzymes in response to oxidants. *The Journal of Biological Chemistry*.

[50] Liochev S. I., Fridovich I. (2010). Mechanism of the peroxidase activity of Cu, Zn superoxide dismutase. *Free Radical Biology and Medicine*.

[51] Acton S. L., Kozarsky K. F., Rigotti A. (1999). The HDL receptor SR-BI: a new therapeutic target for atherosclerosis?. *Molecular Medicine Today*.

[52] Abate N. (2000). Obesity and cardiovascular disease: pathogenetic role of the metabolic syndrome and therapeutic implications. *Journal of Diabetes and Its Complications*.

[53] Yamashita S., Hirano K.-I., Sakai N., Matsuzawa Y. (2000). Molecular biology and pathophysiological aspects of plasma cholesteryl ester transfer protein. *Biochimica et Biophysica Act—Molecular and Cell Biology of Lipids*.

[54] Beisiegel U. (1996). New aspects on the role of plasma lipases in lipoprotein catabolism and atherosclerosis. *Atherosclerosis*.

[55] Zambon A., Hokanson J. E., Brown B. G., Brunzell J. D. (1999). Evidence for a new pathophysiological mechanism for coronary artery disease regression. Hepatic lipase-mediated changes in LDL density. *Circulation*.

[56] Park J.-G., Oh G. T. (2011). The role of peroxidases in the pathogenesis of atherosclerosis. *BMB Reports*.

[57] Beer M., Seyfarth T., Sandstede J. (2002). Absolute concentrations of high-energy phosphate metabolites in normal, hypertrophied, and failing human myocardium measured noninvasively with ^31^P-SLOOP magnetic resonance spectroscopy. *Journal of the American College of Cardiology*.

[58] Conway M. A., Allis J., Ouwerkerk R., Niioka T., Rajagopalan B., Radda G. K. (1991). Detection of low phosphocreatine to ATP ratio in failing hypertrophied human myocardium by 31P magnetic resonance spectroscopy. *The Lancet*.

[59] Tian R., Nascimben L., Kaddurah-Daouk R., Ingwall J. S. (1996). Depletion of energy reserve via the creatine kinase reaction during the evolution of heart failure in cardiomyopathic hamsters. *Journal of Molecular and Cellular Cardiology*.

[60] Kato T., Niizuma S., Inuzuka Y. (2010). Analysis of metabolic remodeling in compensated left ventricular hypertrophy and heart failure. *Circulation: Heart Failure*.

[61] Lei B., Lionetti V., Young M. E. (2004). Paradoxical downregulation of the glucose oxidation pathway despite enhanced flux in severe heart failure. *Journal of Molecular and Cellular Cardiology*.

[62] Hafstad A. D., Nabeebaccus A. A., Shah A. M. (2013). Novel aspects of ROS signalling in heart failure. *Basic Research in Cardiology*.

[63] Marín-García J., Akhmedov A. T., Moe G. W. (2013). Mitochondria in heart failure: the emerging role of mitochondrial dynamics. *Heart Failure Reviews*.

[64] Chabrashvili T., Tojo A., Onozato M. L. (2002). Expression and cellular localization of classic NADPH oxidase subunits in the spontaneously hypertensive rat kidney. *Hypertension*.

[65] Zou A.-P., Li N., Cowley A. W. (2001). Production and actions of superoxide in the renal medulla. *Hypertension*.

[66] Raij L. (2008). Nitric oxide and cardiovascular and renal effects. *Osteoarthritis and Cartilage*.

[67] Ola M. S., Berkich D. A., Xu Y. (2006). Analysis of glucose metabolism in diabetic rat retinas. *The American Journal of Physiology—Endocrinology and Metabolism*.

[68] Tiganis T. (2011). Reactive oxygen species and insulin resistance: the good, the bad and the ugly. *Trends in Pharmacological Sciences*.

[69] Burne-Taney M. J., Rabb H. (2003). The role of adhesion molecules and T cells in ischemic renal injury. *Current Opinion in Nephrology and Hypertension*.

[70] Goh C. Y., Vizzi G., De Cal M., Ronco C. (2011). Cardiorenal syndrome: a complex series of combined heart/kidney disorders. *Contributions to Nephrology*.

[71] Rosner M. H., Ronco C., Okusa M. D. (2012). The role of inflammation in the cardio-renal syndrome: a focus on cytokines and inflammatory mediators. *Seminars in Nephrology*.

[72] Virzì G. M., Torregrossa R., Cruz D. N. (2012). Cardiorenal syndrome type 1 may be immunologically mediated: a pilot evaluation of monocyte apoptosis. *Cardiorenal Medicine*.

[73] Kielar M. L., John R., Bennett M. (2005). Maladaptive role of IL-6 in ischemic acute renal failure. *Journal of the American Society of Nephrology*.

[74] Rabb H. (2006). Immune modulation of acute kidney injury. *Journal of the American Society of Nephrology*.

[75] Ramesh G., Reeves W. B. (2004). Inflammatory cytokines in acute renal failure. *Kidney International*.

